# Frugal innovation in a crisis: the digital fabrication maker response to COVID‐19

**DOI:** 10.1111/radm.12446

**Published:** 2020-12-03

**Authors:** Lucia Corsini, Valeria Dammicco, James Moultrie

**Affiliations:** ^1^ Institute for Manufacturing 17 Charles Babbage Cambridge CB3 0FS UK

## Abstract

The rapid spread of COVID‐19 has led to a global shortfall in essential items, turning many countries into resource‐constrained environments. In response, an unprecedented number of do‐it‐yourself hobbyists (i.e. makers) have started to use digital fabrication tools to produce critical items. These bottom‐up communities are mobilising as part of a global movement to produce innovative solutions to much‐needed items, such as face masks, face shields and ventilators. As these individuals tackle widespread resource constraints, the conceptual lens of frugal innovation becomes highly relevant to study how these solutions developed. Frugal innovation is a type of resource‐constrained innovation that refers to the practice of doing more with less, for more people. In this study, we present two instrumental case studies of maker projects that use digital fabrication to tackle COVID‐19. The first case study is from Italy (a High Income Country) and the second is from India (a Lower Middle Income Country). We analyse the frugality of these cases and highlight their similar approaches. In doing so, we suggest that current theories of frugal innovation can be expanded to new geographical and technological contexts. We put forward that frugal innovation is an important strategy in crisis response beyond emerging markets and that digital fabrication can be considered as an important frugal innovation enabler, both in its ability to produce frugal solutions and to support distributed networks of innovation actors. This study advances knowledge on how frugal innovation unfolds in the Maker movement. It is among one of the first studies to connect the domains of makers and frugal innovation, and the paper concludes by identifying several promising areas for further research.

## 1 Introduction

The rapid spread of COVID‐19 is placing strain on global supply chains (López‐Gómez et al., 2020). Widespread disruption has resulted in shortages of critical items, including Personal Protective Equipment (PPE) (e.g. face masks), clinical equipment (e.g. ventilators) and diagnostics (e.g. nasal swabs) (Chagas et al., 2020). The failure of traditional industry to meet the demand for critical items has seen an unprecedented response from the maker community (Pearce, 2020). These informal networks of innovators are cooperating to produce urgently needed items for COVID‐19 (Corsini et al., 2020b).

Although this is not the first time that makers and makerspaces have played a role in crisis response (Corsini et al., 2019; Corsini and Moultrie, 2019, 2020), this is the first time that the maker community is responding in such numbers. Prior to COVID‐19, research on 3D printing in the humanitarian sector identified a handful of promising projects (Corsini et al., 2020a). Yet, since 28th March 2020 the Facebook group, Open Source Medical Supplies claims to have produced over 7.2 million units of medical items (‘Open Source COVID19 Medical Supplies’, 2020). Whilst an exact definition of ‘makers’ has been notoriously vague in the literature, we consider makers as both formally trained and self‐taught individuals that work with ‘open, peer‐to‐peer, distributed and Do It Yourself (DIY) approaches in a collaborative way… for cultural change, educational and social purposes, beside entrepreneurial ones’ (Menichinelli and Schmidt, 2020).

The Maker movement has emerged in the last decade as an umbrella term to signify the variety of actors who value a DIY culture of making, repairing and hacking (Dougherty, 2012). Although these individuals have always existed, the proliferation of digital fabrication tools (3D printing, laser cutting and CNC milling) and emergence of fabrication workshops (including FabLabs, makerspaces and hackerspaces) has renewed interest in these makers and even led to claims of a new ‘industrial revolution’ being driven by such actors. More precisely, Anderson (2012) characterises these makers by: (1) their use of digital desktop tools to design and prototype artefacts; (2) their culture of open, collaborative and peer production; and, (3) their use of digital fabrication tools, spaces and services to produce such artefacts.

In this study, we analyse the digital fabrication responses of the maker community to COVID‐19 through the lens of frugal innovation. As cases of COVID‐19 continue to rise around the world and the crisis becomes more protracted (BBC News, 2020), resource scarcity is becoming an increasingly pressing issue (Mannelli, 2020). Relevant to this new global landscape, is the conceptual lens of frugal innovation. Frugal innovation is often described as ‘an ability to do more with less’ (Radjou et al., 2012) and typically refers to ‘good enough, affordable products that meet the needs of resource‐constrained consumers’ (Zeschky et al., 2014). An increasingly popular concept in the last decade, it has predominantly been used to study entrepreneurship in low‐income regions in South Asia (Agarwal et al., 2017). To this extent there are a paucity of studies outside the geographical boundaries of India and China. In this study, we consider the COVID‐19 crisis as an external shock which is turning many high income regions into resource‐constrained environments. Hence, we question whether the boundaries of frugal innovation might be expanded to new contexts. This research expands knowledge on how digital fabrication is changing the innovation process (Corsini and Moultrie, 2017).

First, we present the state of the art on frugal innovation, summarising the key characteristics that define its process, outcome and actors. We also summarise existing knowledge on frugal innovation and the Maker movement. Second, we explain the case study methods. Third, we use the frugal characteristics to analyse two instrumental case studies in Italy and India of makers’ digital fabrication responses to the COVID‐19 crisis. In doing so, we primarily focus on how the Maker movement concept fits within the frugal innovation paradigm. We expand on existing theory on frugal innovation, by suggesting that it can play a role in any resource constrained society and that digital fabrication is an important tool for the frugal innovator.

## 2 Literature on frugal innovation

Weyrauch and Herstatt (2017) and Khan (2016) have analysed the characteristics of frugal innovation in two recent and highly cited papers. We focused on synthesising these contributions, using snowballing to identify other highly cited, seminal works to review the key attributes of the frugal concept. This include academic journals and conference papers, as well as books and book chapters. After reviewing the existing literature, it appeared clear that the frugal concept has been studied to describe both a particular innovation process and outcome, as well as a type of actor able to carry out the frugal innovation from start to end.

Table 1 provides a summary of these attributes, with key citations highlighted to evidence each attribute. These citations are not intended to be exhaustive, however, provide an overview of some of the important academic contributions in the field. We briefly discuss some of these key themes below to help guide the reader.

**Table 1:** Summary of the key attributes of frugal innovation and frugal innovators

		Key attributes of frugal innovations & innovators	Key citations
Frugal innovations	Innovation outcomes	Low‐cost, affordable, cost‐effective	Angot and Plé (2015), Hossain (2017), Rao (2013), Zeschky et al. (2014)
		Oriented towards emerging markets	Zeschky et al. (2014)
		Good enough performance	Agarwal et al. (2017), Radjou et al. (2012)
		Simplicity, ease of use	Radjou et al. (2012), Rao (2013)
		Enables new applications	Zeschky et al. (2014)
		User‐centred	Pansera and Sarkar (2016), Zhang (2018)
		Sustainable, eco‐friendly	Angot and Plé (2015), Brem and Wolfram (2014), Radjou and Prabhu (2014)
		Disrupts incumbents	Brem and Wolfram (2014), Rao (2013)
	*Innovation process*	Uses low‐tech (non‐digital)	Agarwal et al. (2017), Radjou et al. (2012)
		Design based on constraints	Pansera and Sarkar (2016), Prabhu (2017), Zhang (2018)
		Uses local resources	Bhatti and Ventresca (2013), Gibson and Shukla (2016)
		Local production	Zeschky et al. (2014)
		Large‐scale outsourcing	Zeschky et al. (2014)
		Uses new distribution models	Sharma and Iyer (2012)
Frugal innovators		Quick thinkers and doers	Agarwal et al. (2017), Radjou et al. (2012)
		Opportunistic	Radjou et al. (2012)
		Have ‘innovative friends’/social capital	Radjou and Prabhu (2014)
		‘Make do with less’/DIY bricolage attitude	Prabhu and Gupta (2014), Radjou et al. (2012)

### 2.1 Frugal innovations and processes

At its heart, frugal innovation is about doing more, for less, for more people (Prabhu, 2017). A frugal product is characterised by certain design and production features. First and foremost, frugal innovations make minimal use of resources since they are tailored for environments with poor infrastructures (Zeschky et al., 2014). The new products are developed using resources that are more readily available and low‐cost (Sharma and Iyer, 2012). The minimal use of resources results not only in the economising of components and local raw materials, but also in the creation of simpler designs (Rao, 2013; Pradel and Adkins, 2014). This cost and resource efficiency/minimisation can be achieved throughout different stages of the product life cycle (Pradel and Adkins, 2014), as frugal innovations are sourced and produced locally (Zeschky et al., 2014) and often have to employ new distribution models to overcome logistics voids imposed by the resource‐constraint environment (Sharma and Iyer, 2012).

Due to the design constraints imposed by the paucity of resources, these innovations developed frugally are often less technologically advanced than their more sophisticated counterparts that have been produced under traditional innovation methods (Radjou et al., 2012). Despite their lower level of sophistication, frugal innovations are found to display good‐enough performance and functionality for their intended scope (Agarwal et al., 2017).

The above characteristics result in the creation of a product that is intrinsically sustainable and eco‐friendly (due to the minimisation of costs and resources), user‐centred and inclusive of segments of the populations that are usually underserved (Kahle et al., 2013). Because frugal innovations exist to fill institutional voids in fast and targeted ways, current incumbents are likely to be disrupted by frugal innovators (Rao, 2013; Brem and Wolfram, 2014).

### 2.2 Frugal innovators

Frugal innovation has been studied not only by looking at the peculiarities of its process, but also by looking at the attitude, beliefs and philosophy of making that characterises its innovators (Bhatti and Ventresca, 2013). Frugality is about ‘doing more with less’ (Radjou et al., 2012; Prabhu and Gupta, 2014), it is not just about eliminating waste, but also embracing reduction and short‐term sacrifices to achieve long‐term goals (Bhatti and Ventresca, 2013). Frugal innovators are characterised as quick thinkers and doers (Radjou et al., 2012; Agarwal et al., 2017), able to turn the resource‐constraints of the environments into opportunities (Radjou et al., 2012). Frugality is, therefore, conceptualised as a distinctive behavioural trait that relates to bricolage behaviour and effectuation behaviours i.e. affordable loss and flexibility (Sarasvathy, 2001). Finally, frugal innovators have been found to have higher degrees of social capital and ‘innovative friends’ in their network (Radjou and Prabhu, 2014).

### 2.3 Frugal innovation and the Maker movement

To date, there is barely any scholarship that examines the relationship between frugal innovation and makers. Apart from some academic literature on the related concept of bricolage and the Maker movement (Rumpala, 2014; Beltagui et al., 2019), studies on frugal innovation and makers have remained largely isolated.

Among sparse literature, Prabhu (2017) observes that the Maker movement is an example of the sharing economy that acts as a ‘demand‐side driver of frugal innovation’. Elsewhere, there are a handful of references to frugal innovation and the Maker movement, yet, this research does not explores these linkages in any detail (Dandonoli, 2013; Wohlfart et al., 2016). For example, Corsini and Moultrie (2018) discuss how digital fabrication increases opportunities for improvisation through making, and Seo‐Zindy and Heeks (2017) suggest that aspects of frugal innovation are compatible with the ‘making‐do’ culture associated with digital fabrication networks, yet, they offer little substantive investigation.

Research related to frugal innovation and digital fabrication is equally limited. In part, this could be explained by the perceived ‘low‐tech’ nature of frugal innovation (Radjou et al., 2012). Among very few studies Maric et al. (2016) suggest that 3D printing itself could be viewed as a frugal technology. In a book chapter Gibson and Shukla (2016) put forward that 3D printing offers the possibility for more eco‐friendly and frugal solutions through the localisation of supply chains. Elsewhere, there is some literature that briefly references 3D printing and frugal innovation (Radjou and Euchner, 2016; Agarwal et al., 2017) and Smith et al. (2013) list frugal innovation as a theory that could help to conceptualise grassroots digital fabrication.

Given the paucity of existing knowledge, yet, the apparent relevance of frugal innovation to digital fabrication/maker practices, we set out the following research questions: how can frugal innovation help us to understand how innovation happens in the Maker movement? How can the Maker movement response to COVID‐19 contribute to theory on frugal innovation? By using frugal innovation as a conceptual lens to analyse digital fabrication maker responses to COVID‐19, we seek to advance theory on frugal innovation, and formally connect the Maker movement to the frugal innovation paradigm.

## 3 Methods

With a view to elaborate and examine the construct of frugal innovation, we use an instrumental (theory‐based) case study approach based on Palinkas et al. (2015). In an instrumental case study, a particular case is selected to expand on a theoretical construct, which in this study is the frugal innovation concept. Prior to selecting the cases, the authors reviewed social media, news reports and open databases (e.g. ‘COVID‐19‐Solutions’, 2020) to better understand the landscape of digital fabrication maker responses to COVID‐19. This review took place during the early stages of the coronavirus pandemic from 15th March to 7th April 2020. At the time, few of the numerous Maker responses had been fully implemented and had reached significant distribution within countries.

Hence, in order to narrow down the potential case studies, we set out the following criteria. First, we searched for highly prominent cases that had received significant attention in the press. Second, we looked for cases that had successfully implemented solutions. Cases that had not reached a level of maturity to establish successful implementation were excluded. Third, we pragmatically searched for cases for which it was possible to identify a key point of contact. For example, highly dispersed networks such as the Facebook group ‘Open Source Medical Supplies’ were excluded on this basis. Fourth, we wanted to select case studies that together represented the geographical diversity of maker responses. This inclusion/exclusion criteria led to the identification of two suitable cases: The first case (Isinnova) is from a High Income Country (HIC) and the second case (M‐19 Collective) is from a Lower Middle Income Country (LMIC).

In an effort to build up rich case studies, data were collected from multiple sources including: news articles, documents, organisational websites and social media. Secondary data were used to build up a narrative of the case studies and to inform our initial understanding of their relevance to the frugal innovation concept. These data helped to guide the semi‐structured interviews, which were conducted by the first author via phone between 15 and 18th April 2020, with practitioners at both case studies (Cristian Francassi, CEO at Isinnova; Alessandro Romaioli, Engineer at Isinnova; Vaibhav Chhabra, Co‐founder at Maker’s Asylum). The interviews covered a range of topics including: project description, motivations, actors involved, key enablers and constraints. The interviews lasted between 30 and 40 min and were all recorded with the participants’ consent. All the interviews were transcribed verbatim afterwards. These transcripts were imported into MAXQDA and a code hierarchy was created in the data analysis software based on the attributes of frugal innovation presented in Table 1. Following guidelines to qualitative coding by Saldaña (2015), line by line coding of the interview data was conducted to check whether and how these innovation/innovator attributes appeared in the case studies (see Appendix Table A1). For example, ‘*We kept the price pretty much as low as possible’* was coded as ‘low‐cost, affordable, cost effective’. ‘*Initially we were making the shields in acrylic, but then soon we ran out of acrylic … one of the co‐founders suggested, "Why don't we use foam board?”… it made so much sense because foam board is available in each and every stationary shop across the world’* was coded as ‘uses local resources’. Secondary data were used to complement and validate this analysis. These data were particularly valuable because of the fast‐moving nature of the COVID‐19 response, which meant that the case studies were evolving even as the analysis was being undertaken.

## 4 Results

The following section first describes the case of Isinnova, presenting it as an example of a frugal innovation from a High Income Country (HIC). It then introduces the case of M‐19 Collective as an example of a frugal innovation from a Lower Middle Income Country (LMIC). Table 2 compares the case studies to illuminate their frugal characteristics.

**Table 2:** Comparison of the frugal characteristics identified in the case studies

		Key attributes of frugal innovations & innovators	ISINNOVA – CASE 1	M‐19 COLLECTIVE – CASE 2
Frugal innovations	Innovation outcomes	Oriented towards emerging markets	*X*	✓
		Simplicity, ease of use	✓	✓
		Low‐cost, affordable, cost‐effective	✓	✓
		Good enough performance	✓	✓
		Enables new applications	✓	✓
		User‐centred	✓	✓
		Sustainable, eco‐friendly	*?*	✓
		Disrupts incumbents	✓	✓
	Innovation process	Uses low‐tech (non‐digital	*X*	*X*
		Design based on constraints	✓	✓
		Uses local resources	✓	✓
		Local production	✓	✓
		Large‐scale outsourcing	✓	✓
		Uses new distribution models	✓	✓
Frugal innovators		Quick thinkers and doers	✓	✓
		Opportunistic	✓	✓
		Have ‘innovative friends’/social capital	✓	✓
		‘Make do with less’/DIY bricolage attitude	✓	✓

### 4.1 Frugal innovation in a High Income Country: the case of Isinnova

Isinnova is an Italian rapid prototyping start‐up that initially received widespread acclaim for their work to 3D print life‐saving valves for ventilators in Italy. When replacement valves for the ventilators could not be sourced by the manufacturer in time, the Hospital of Brescia contacted the local newspaper to put out a call for help. The editor of the newspaper was familiar with the work of FabLab Milano and after contacting them to confirm that it would be possible to 3D print a valve, reached out to Isinnova who were based in Brescia.

**Figure 1:**
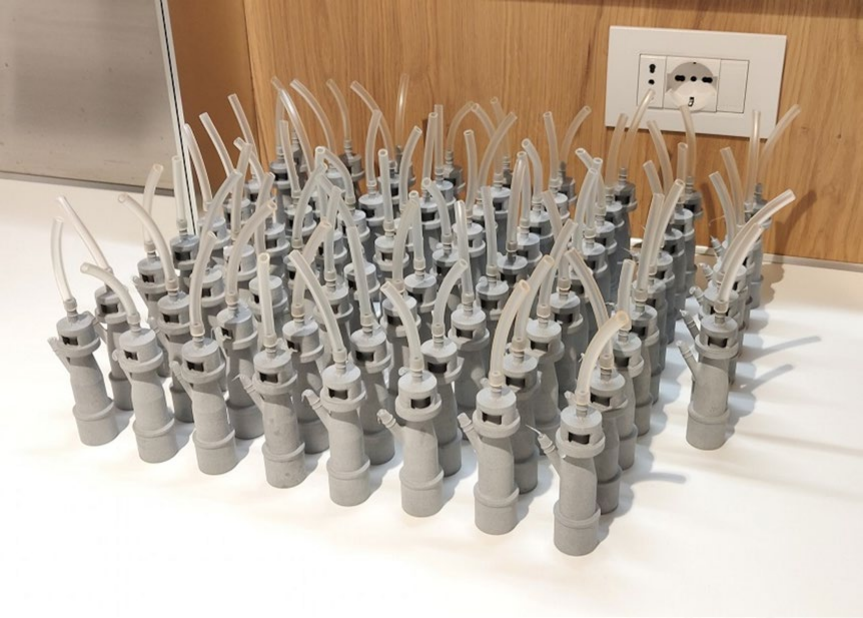
3D printed ventilator valves © Isinnova. [Colour figure can be viewed at wileyonlinelibrary.com]

In less than a day, Cristian Francassi (CEO at Isinnova) and Alessandro Romaioli (Engineer at Isinnova) were able to reverse engineer, print and distribute valves for ventilators. Once they visited the hospital to test that their initial prototype worked, they began to scale up production by leveraging the local maker ecosystem of 3D printers. With each valve taking around 30 min to print, they were able to produce a total of 100 life‐saving valves in under 24 hr (see Figure 1).

Their response is typical of the frugal innovator’s ability to reframe adversity and adapt to changing circumstances. Isinnova were able to quickly apply their experience using 3D printing to a new application – in this case translating their background in rapid prototyping to medical device innovation. In this case, resource constraints are not simply to do with a lack of materials but a pressing lack of time:

The first challenge was the time because we had to do it in a fast way that we could not imagine before. We had to redesign, reprint and distribute in less than 24 hr… Just imagine that if you produce any medical product you need about 8 to 12 months to get a certification in Italy. So doing it in a 24 hr even if you don't have a certification, it's unthinkable. – Alessandro Romaioli, Engineer at Isinnova

In this case, it is clear that social capital is a key enabler for the frugal innovator. Isinnova’s position within the local innovation ecosystem in Brescia meant that they were well placed to respond to the initial call for help from the local hospital. Their own relationships with existing makers and manufacturers also meant that they were able to call on their ‘innovative friends’ (Radjou and Prabhu, 2014) to scale‐up production in a very short period of time. This idea of being ‘in the right place at the right time’ is crucial for the frugal innovator, however, it underlies a deeper reality that serendipity and social capital work hand in hand. Whilst Isinnova contend that their success was a result of luck, a closer examination of this case highlights that the frugal innovator makes their own luck by leveraging their social capital and exploiting immediate opportunities.

It was a matter of luck for sure. We just had the intention to do the best project possible to try to help people. – Cristian Fracassi, CEO Isinnova

**Figure 2:**
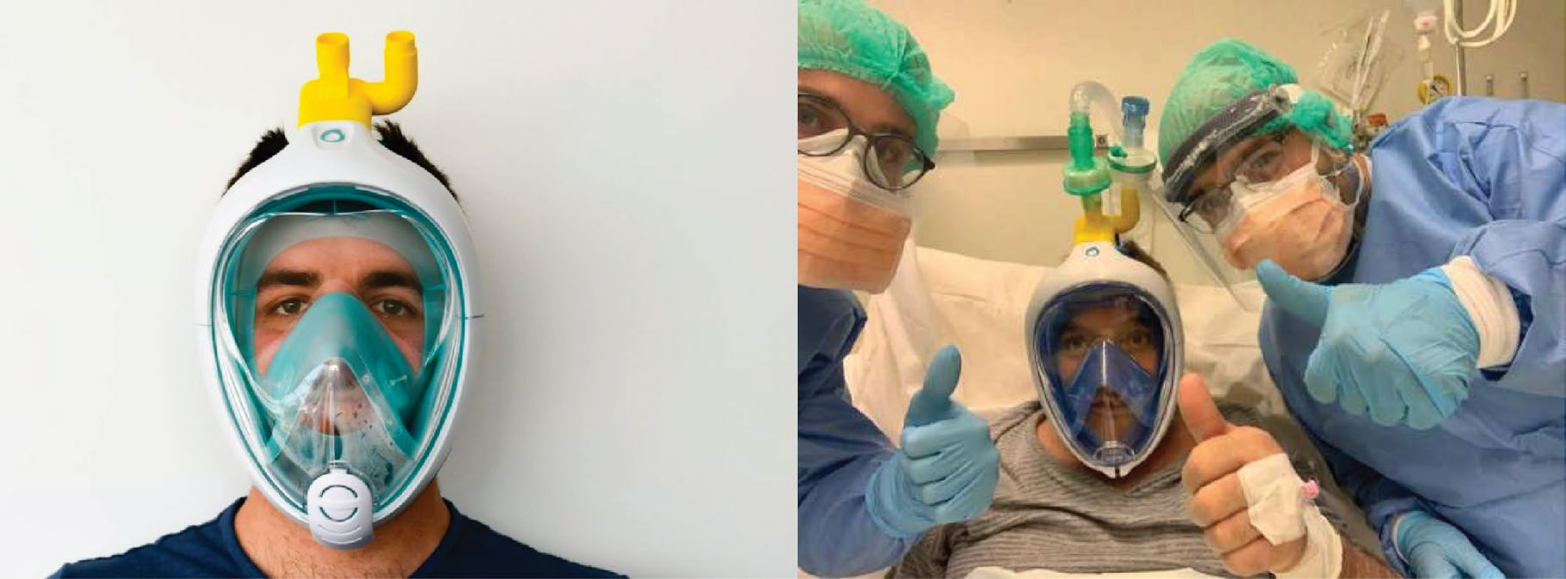
Scuba mask adapted into a CPAP mask using a 3D printed Charlotte Valve © Isinnova. [Colour figure can be viewed at wileyonlinelibrary.com]

Following the widespread publicity about this initiative, a retired doctor Renato Favero contacted Isinnova with an idea for another design. His experience led him to anticipate widespread shortages of CPAP (Continuous Positive Airway Pressure) masks that are needed to provide assisted breathing. He wondered if a full‐face scuba diving mask could be repurposed to this effect. A collaboration was quickly established between Renato Favero and the team at Isinnova, with support from the local hospital in Brescia. The team contacted Decathlon, the producer of the scuba diving mask who provided the design files of the scuba mask for the team to develop a solution. Within ten hours, they had designed and printed a ‘Charlotte valve’, an attachment that could be added to the scuba mask to repurpose it into a fully functioning CPAP mask (see Figure 2).

Once the device had been successfully tested at the local hospital, Isinnova decided to quickly patent the designs and make them openly accessible. In March 2020, over 1000 masks were distributed to more than 50 hospitals in Italy. The design for the Charlotte valve has been downloaded over 2.5 million times by makers across the world. Isinnova reports that over 50,000 masks have been manufactured in Brazil alone, with other initiatives taking place in the US, Canada, Brazil, Morocco, Tunisia, Spain, Singapore and Australia. Their designs can be freely used or adapted to work with other types of mask available on local markets. By anticipating the need for respiratory masks before the demand for CPAP devices became urgent, Isinnova were able to rapidly develop a solution just in time. This ‘closeness’ to the problem is typical of the user‐centred nature of frugal innovations. Their ability to respond quickly to the needs of hospitals is what sets them apart from other well‐intended maker initiatives that have struggled to reach implementation.

This case also marks an interesting departure from mainstream examples of frugal innovation, which predominantly focus on developing economies and Bottom of the Pyramid (BOP) markets. It reinforces the proposition that frugal innovation is a globally relevant concept and highlights the increasing likelihood of frugal innovations in times of crisis. Isinnova explains that in ‘normal’ times, a retrofitted scuba mask using 3D printing would not meet required standards, however, given the urgent demand and lack of alternatives, the solution was welcomed by practitioners.

The hospital cannot use 3D printed pieces in normal conditions but in this emergency the hospital is allowed to do whatever they can to try to help… say there were 100 people in need of oxygen and the hospital had only 30 masks and so there were 70 people there without the oxygen. We offered the hospital a second chance to try to treat people, even if it hasn't been certified. A doctor told us, “If I have to leave a patient without oxygen or try to treat him with a 3D printed part, what do you think I will do? Of course, I will try to do whatever I can to try to help him.” – Cristian Francassi, Engineer at Isinnova

Finally, this case illuminates a new form of reverse innovation, a key concept related to frugal innovation. Typically reverse innovation refers to the process by which products designed in LMICs are then diffused as low‐cost solutions in HICs. In this example, we find that the new paradigm of distributed manufacturing can give rise to frugal innovations being shared from HICs to LMICs. It underlines the potential for frugal innovation to leverage new distribution models enabled by digital fabrication.

So other people from around the world download our file and work on it and improve it. For now, we have found 11 different Charlotte valve files with different form. – Cristian Francassi, CEO at Isinnova

We were among the first to start to do something with 3D printing. We gave an example and other people found inspiration in our work. – Alessandro Romaioli, Engineer Isinnova

### 4.2 Frugal innovation in a Lower Middle Income Country: the case of the M‐19 Collective

Maker’s Asylum founded the M‐19 collective to create a single and united response to COVID‐19 from maker initiatives across India. Maker’s Asylum is a makerspace in Mumbai that mainly work on educational and rapid prototyping projects. After facing several projects cancellations due to COVID‐19, the co‐founders of Maker’s Asylum began experimenting with ways to make face shields by hand. They posted videos of their work online and soon started receiving requests from local hospitals. Their initiative exemplifies the attitude of the frugal innovator – tackling uncertainty head on and leveraging adversity.

We have a staff of about 10 people… we were pretty much in tears because we were trying to figure out how we were going to survive this entire thing, because when Mumbai went on lockdown all our international programmes… they all got cancelled. Because all the programmes got cancelled, all the funds were over, so me and one of my colleagues decided to stay at Maker's Asylum instead of going back home… to figure out something to do during this time. So we started living over here and then, after a day, we made a DIY face shield video… we put up a small crowdfunding campaign of 1000 shields to start with. Within a day, we had to change that number to 10,000 shields. – Vaibhav Chhabra, Co‐founder Maker’s Asylum

Maker’s Asylum received a sudden influx of orders for their face shields. They began experimenting with both 3D printing and laser cut versions and set themselves an initial target of making 10,000 face shields. Importantly, they realised that the project’s success relied on developing an affordable product that met the needs of their users. As identified earlier, affordability and user‐centredness are key attributes of frugal innovations.

So while we were making it, we were also talking to a lot of doctors and hospitals. We did a lot of product testing on ourselves first… Every day at Maker's Asylum, every single one of the volunteers has been wearing the shield themselves. So we're actually testing these shields and wearing them for at least eight hours a day, all of us, to make sure that what we're giving out to the hospitals is good enough. – Vaibhav Chhabra, Co‐founder Maker’s Asylum

Adopting a ‘quick thinking and doing’ frugal attitude, they distributed their first face shields in less than a week. After receiving positive feedback from hospitals, word of mouth began to spread news of their work and the number of requests for face shields began to rise steadily. They also received several endorsements from government ministers, which helped to build their reputation.

**Figure 3:**
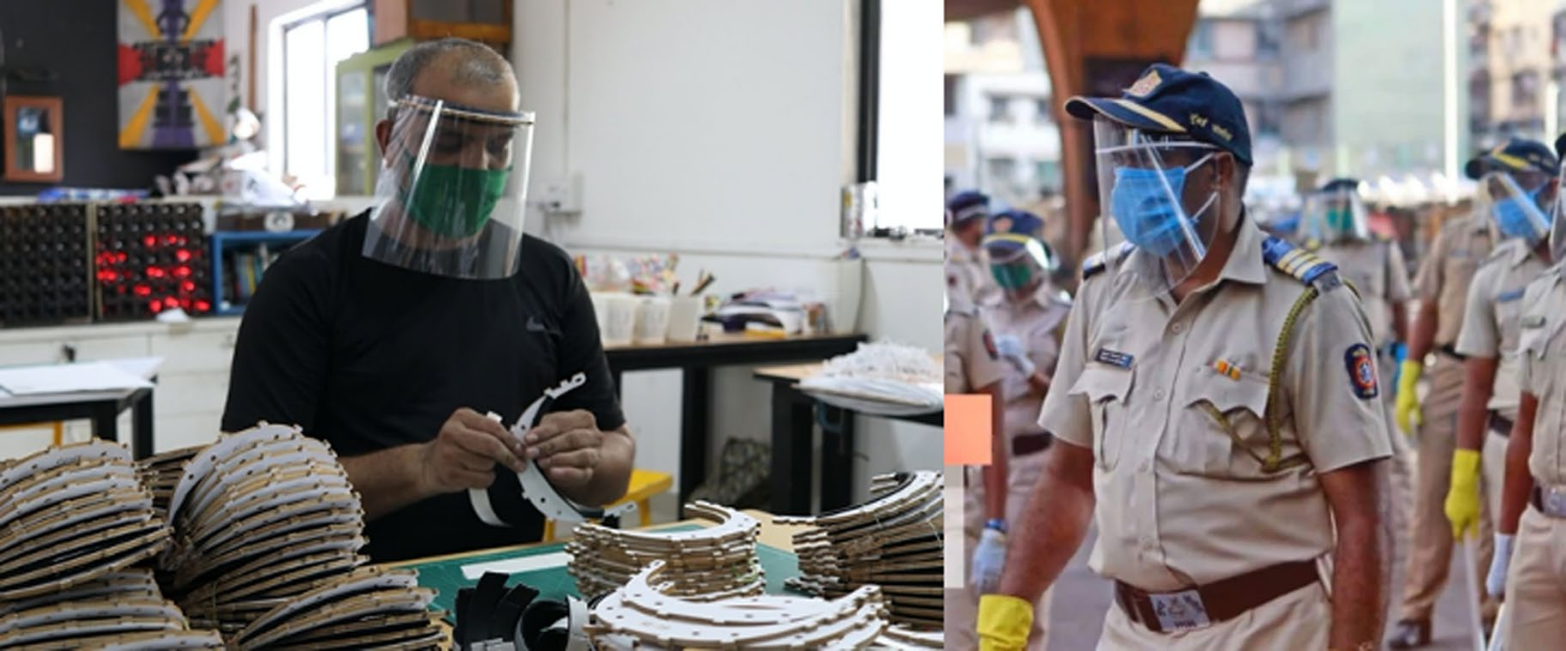
Assembling face shields (left), Mumbai Police wearing face shields produced by the M‐19 Collective (right) © M‐19 Collective. [Colour figure can be viewed at wileyonlinelibrary.com]

As demand grew, Maker’s Asylum set a new target to manufacture 100,000 face shields. They quickly realised that they needed to join forces with other tech‐shops and makerspaces across India. They started talking to other labs and sharing instructional videos about their designs and production lines. Recognising that a unified maker response would amplify impact, they established the M‐19 Collective. Since then, over 42 makerspaces have joined the collective and over 1,000,000 face shields have been produced and distributed to front line workers (see Figure 3).

Maker’s Asylum also spotted another opportunity to further decentralise their production. When they realised that they were producing the face shields faster than they could be assembled, they spotted the untapped potential of medical students who were quarantined in their residence at local hospitals. Maker’s Asylum then switched production to DIY kits, as these unoccupied medical students volunteered to assemble and sanitise the face shields on‐site at the hospitals. This innovative approach to distribution is typical of frugal solutions.

Inside the hostels are the first and second year students who are sitting over there, waiting, quarantined and they don't have anything to do and they want to do something to help out with the doctors… they get the DIY kit and they assemble it… that completely localises the assembly line…Now, if I start giving it to households to assemble, that’s not good of an idea… You can't quality control. But inside a medical hostel, you can do that, because there's a doctor who can supervise it – Vaibhav Chhabra, Co‐founder Maker’s Asylum

**Figure 4:**
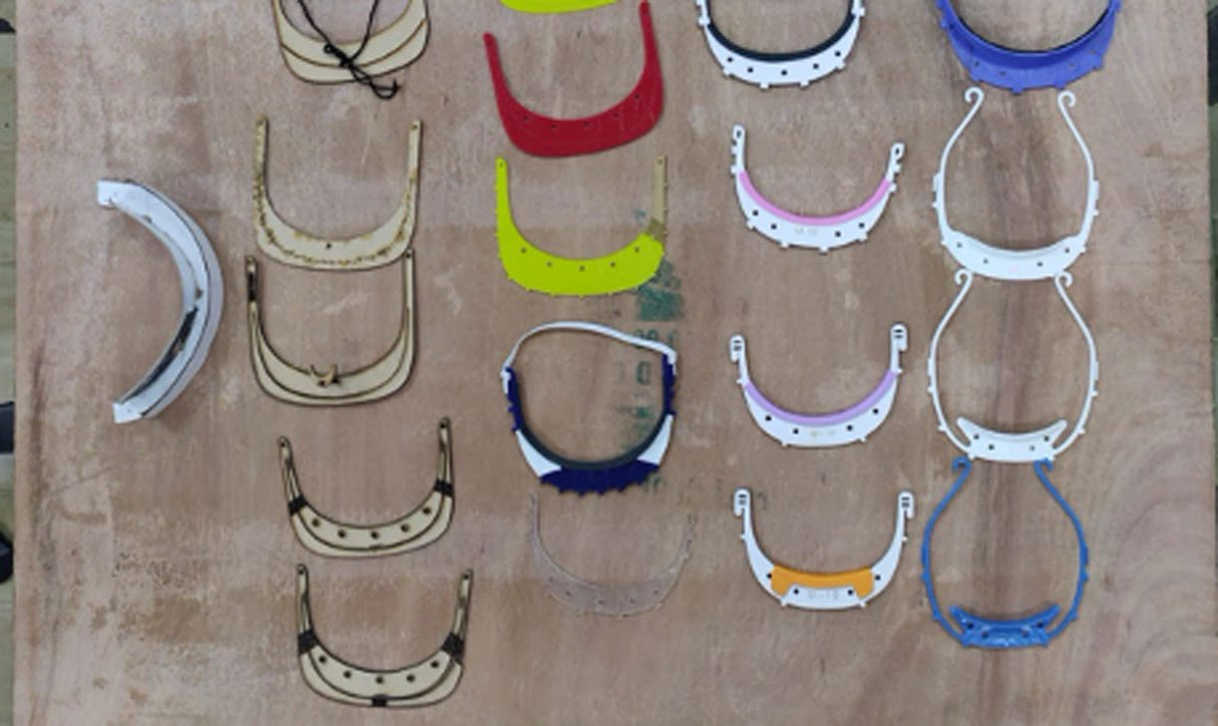
Multiple design iterations of headband for the face shields © M‐19 Collective. [Colour figure can be viewed at wileyonlinelibrary.com]

All of Maker’s Asylum’s designs are freely available on GitHub, an open design repository. However, not every makerspace in the collective is making the same design. Working in a frugal way, each makerspace adapts designs according to local needs and availability of materials. Since the initiative started, Maker’s Asylum have developed over 21 different design iterations (see Figure 4). For example, the original face shields were using acrylic sheets for the headpieces, however, after facing material shortages they began using foam board, a material readily available at local stationary shops. When they were unable to source PET sheets for the visors, they started using affordable and commonly available Over Head Projector (OHP) sheets. What unites the M‐19 Collective is not a single design, but a single mission and approach.

It's not about the same design. Every lab is making multiple design. What we're trying to do is just combine the initiative of all of the labs, to go out with one voice, the fact that all these smaller hubs across the country can take whichever design, whatever that works, that's approved, as long as it's approved by doctors and other people. We're able to serve, give them out, and have an impact as a collective then. That was important. – Vaibhav Chhabra, Co‐founder Maker’s Asylum

To their success, Maker’s Asylum have not just developed a face shield that constitutes a frugal innovation, but they have truly embodied the attitude of frugal innovators. What is most striking about this case, is the group’s ability to continuously exploit gaps in the system. A final example is Maker’s Asylum’s creation of a new barter economy. Realising the value of their face shields to street food vendors, they have effectively started exchanging face shields for food for the volunteers working at the makerspace.

The currency at Maker's Asylum at the moment is shields… they give us food, instead we get them shields. – Vaibhav Chhabra, Co‐founder Maker’s Asylum

## 5 Discussion

As COVID‐19 is globally expanding resource constraints, frugal innovation was selected as a relevant lens to investigate the activities of the digital fabrication maker community. This study has enriched our understanding of how innovation unfolds in the Maker movement, as well as helping to address the under‐development of the frugal innovation concept.

First, by introducing frugal innovation, we have added a new theoretical perspective to studies on the Maker movement. We have explicitly shown how the digital fabrication maker community is well‐suited to dealing with resource constraints. In addition, we have highlighted that makers demonstrate many of the same characteristics of frugal innovators. What is common to makers and frugal innovators is their ability to respond quickly to problems as they arise. They work in opportunistic ways, exploiting their own social networks to develop solutions. In the cases analysed, the makers tackled problems within the constraints of the existing system, in what has recently been conceptualised in social innovation literature as ‘systems hacking’ (Savaget et al., 2019). To date, studies on the maker community have tended to focus on shared values and beliefs (Katterfeldt, 2012) and less attention has given to the characteristics of makers themselves. This research raises the prospect that engagement with the Maker movement can help to cultivate the mindset of the frugal innovator, particularly in regions where there is not a strong cultural legacy of frugal innovation. Also considering Smith's (2017) proposition that makerspaces can help to anticipate new material cultures, managers might consider how their employees could engage with new fabrication spaces and emerging maker practices and in order to develop the skills for resource‐constrained innovation.

Second, this study has expanded current theories of frugal innovation to new geographical and technological contexts. It has been said that frugal innovation ‘is in a state of infancy from a theoretical perspective’ (Hossain, 2018). This study has questioned the prevailing logic that frugal innovation is primarily concerned with low‐tech (non‐digital) solutions (Prabhu and Jain, 2015; Agarwal et al., 2017; Winterhalter et al., 2017). By presenting both case studies as frugal innovations, we have clearly demonstrated the role that digital fabrication can play in developing frugal solutions. We thus suggest that the ‘high‐tech’ versus ‘low‐tech’ divide that emerges in literature on frugal innovation is misleading and that emphasis should be placed on contextual relativity. In other words, in both case studies digital fabrication tools are *relatively* frugal compared with industrial manufacturing tools, even if they are generally perceived as being ‘high‐tech’ digital solutions.

Our findings suggest that it is not simply the intrinsic capabilities of digital fabrication, but rather its ability to support geographically distributed maker networks that enables frugal innovation. In both of the cases discussed in this research, their frugality is driven by the ability of makers to locally replicate, adapt and produce these innovations ‐ according to their own needs and constraints. Recognising maker networks as a possible configuration for replicating solutions may well help to address concerns about the scaling‐up of frugal innovations (Bocken et al., 2016). We reflect on this emerging phenomenon as a type of ‘networked frugal innovation’. Future research might expand on these ideas to understand how these frugal maker networks initiate, expand and mobilise under resource‐constraints.

In addition, our case studies have provided much‐needed evidence that frugal innovation is a relevant concept for more than just emerging markets. The Maker movement itself is a geographically dispersed movement, which has recently been called translocal (i.e. globally connected and locally rooted) (Schmidt, 2019). By framing digital fabrication maker responses as frugal innovations, we have demonstrated the relevance of the frugal innovation strategy in new territories outside of the Global South. This pandemic has shown that resource scarcity is not just a result of systemic deprivation (as is found in low‐income regions) but that it can also occur as a result of external shocks such as environmental disasters, economic recessions or public health crises (Doern et al., 2019). Under these conditions, we have highlighted the relevance of the frugal innovations that result from the maker community. Future research might investigate to what extent frugal innovations developed by makers can serve as more than stop‐gap solutions in HICs, and what role frugal maker solutions might play in protracted crises and other stages of crisis response (i.e. recovery, mitigation, preparedness).

## 6 Conclusion

Using the conceptual lens of frugal innovation, this study has helped to shed a light on some of the maker responses to COVID‐19 using digital fabrication. In doing so, we have expanded knowledge on how innovation takes place in the Maker movement, as well as developing theory on frugal innovation.

We analysed two case studies of digital fabrication maker responses to COVID‐19 using the attributes of frugal innovation found in the literature. By framing these cases as frugal innovations, we underlined the parallels between frugal and maker innovation processes and outcomes, as well as identifying many shared attributes of makers and frugal innovators. This research suggests that digital fabrication helps to amplify the work of the frugal innovator, both in its ability to develop frugal solutions and its support of distributed maker networks. We further propose that engagement with the Maker movement can help to cultivate a frugal mindset.

Our findings have also helped to expand the geographical and technological boundaries of frugal innovation theory. We have revealed that frugal innovation is relevant beyond emerging markets. The case studies show that frugal innovation is an important strategy for dealing with crisis in HICs. Even in regions where there is not a strong legacy of frugal innovation, makers are well suited to adopting frugal practices. In addition, we showed that contrary to mainstream accounts that have predominantly focused on low‐tech solutions, digital fabrication (often perceived as ‘high‐tech’ in resource constrained environments) has an important role to play in the development of frugal innovations.

Overall, we believe that this study has uncovered a fruitful research topic that could be developed in several directions. Future research might consider: how the attitudes and behaviours of makers and frugal innovators are in conflict and alignment; how frugal networks of distributed makers emerge and develop; and, how frugal innovation might inform a long‐term strategy for dealing with crisis, beyond stop‐gap solutions.

## Appendix

**Table A1:** Comparing the frugal attributes of the case studies

Key attributes	Isinnova (HIC Context)	M‐19 Collective (LMIC Context)
***Frugal innovations***		
*Simplicity, ease of use*	The ventilator valve and Charlotte valve are each produced from a single printed part, that can easily fit with existing ventilator machines.	Face shield is made of three parts. The headband is made of foam board, the visor is made of PET sheet and it is secured with an elastic band. Easy to assemble.
*Low‐cost, affordable, cost‐effective*	Ventilator valve and Charlotte valve cost less than $1 to produce. Scuba masks were donated by Decathlon.	Each face shield costs less than $0.75
*Good enough performance*	Ventilator valve and Charlotte valve were performance tested at local hospitals. The nature of the crisis meant that hospitals were willing to accept ‘good enough’.	Quality has been validated and accepted by healthcare practitioners.
*User‐centred*	Ventilator valve and Charlotte valve were designed with clinicians and tested at the local hospital.	Face shields were developed and tested with input from local hospitals. Face shields are also used by the production team. New face shields have been developed for infants.
*Sustainable/eco‐friendly*	?	Face shields are reusable, material reduction minimises resource usage.
*Disrupts incumbents*	This is a stop gap solution whilst incumbents cannot respond quickly enough. It signals the potential of distributed (small, localised and decentralised) manufacturing.	21 different versions of the face shield have been developed so far in response to shifting requirements and material constraints.
*Design based on constraints*	Ventilator valve and Charlotte valve are constrained by locally available resources and equipment.	Began improvising by making a face shield by hand before experimenting with 3D printing and then, laser cutting. Self‐described ‘jugaad innovators’.
*Uses local resources*	Makes use of local 3D printing ecosystem. Charlotte valve repurposes readily available scuba masks into a ventilator masks. Charlotte valve can be modified to suit other locally available face masks.	Initially used acrylic for the headpiece but when this became difficult to source locally they started using foam board from local stationary shops. When PET sheeting for the visor became scare they started using OHP sheets.
*Local production*	Designs for Charlotte valve are made openly available to support local adaption and production around the world. Projects operational in Brazil, US, Canada, Morocco, Tunisia, Spain, Singapore, Australia.	Local manufacture of over 1 million face shields at 42 makerspaces across India. This collective has grown steadily since Maker’s Asylum founded the M‐19 collective on 29th March 2020.
*Large scale outsourcing*	To scale up production, production of valves is outsourced to other makers/manufacturers.	To scale‐up production and reduce bottlenecks, collective has started producing DIY face shield kits. These kits are sent to medical students residing in local hospitals, who are responsible for locally assembling face shields on‐site.
*Uses new distribution models*	Distributed manufacturing and distribution to scale up production. Design for the Charlotte valve is freely available on Isinnova’s website.	Distributed manufacturing and distribution to scale up production. All designs are made available on GitHub using CERN Open Hardware License.
***Frugal innovators***		
*Quick thinkers and doers*	Ventilator valve was designed, tested and distributed to hospitals in less than 24 hr. Charlotte valve was designed, tested and distributed within 3 days.	Designed to be comfortable to wear for more than 8 hr. Easy to fit and clean.
*Opportunistic*	Capitalised on being in the right place at the right time. Press about the ventilators valves led to further success – they were contacted by a doctor with an idea to develop the Charlotte valve.	Maker’s Asylum (who initiated the M‐19 collective) faced lots of project cancellations due to COVID‐19. They spotted a gap and starting experimenting with solutions. They realised the value of their work and began bartering with food sellers – swapping face shields for food to support their volunteers.
*High social capital / ‘innovative friends’*	Quickly formed new collaborations with local hospitals and clinicians. Leveraged existing relationships with local makers and incumbent manufacturers to scale up production. Endorsements from the government ministry helped to advance their credentials.	A diverse team of analysts, entrepreneurs and creatives are taking on the role of designers and makers. Mobilising a network of over 300 volunteers across India. Leveraging existing networks and building social capital through endorsements from local and national media, as well as government officials.
*‘Make do with less’/ DIY bricolage attitude*	‘DIY’ attitude helped to reconfigure scuba mask into CPAP ventilator mask.	Using updates on social media to build momentum. Launching an effective crowdfunding campaign to support work – over £28,000 raised in public donations.
